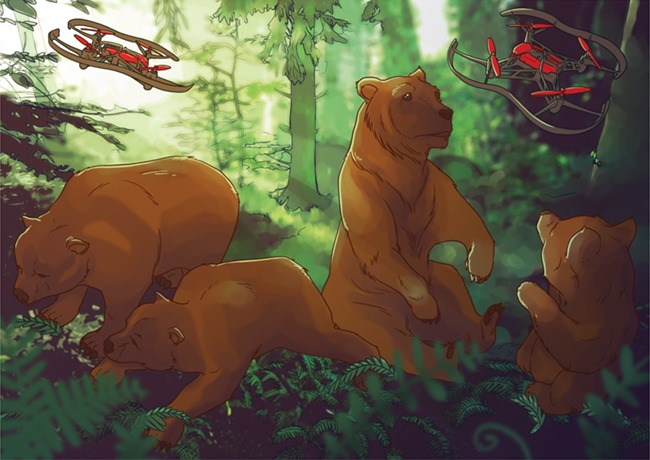# Is droning on making life unbearable?

**DOI:** 10.1093/conphys/coz034

**Published:** 2019-06-17

**Authors:** Gail D Schwieterman

**Affiliations:** Department of Fisheries Science Virginia Institute of Marine Science, William & Mary, Gloucester Point, VA, USA

With the unique ability of remote aircraft systems to hover low to the ground, drones have become an increasingly popular technology used both recreationally and professionally. Limited regulations, rapid technological advances in imaging, and increasing affordability are making drones a new favourite tool for wildlife researchers. However, as anyone who has been near a drone will tell you that loud buzzing sound—imagine horror movie-sized mosquitoes—can be extremely stressful for the unsuspecting.

Indeed, researchers have documented the effect of drone noise on animal stress levels. Wild black bears’ heart rates quadruple when a drone is nearby! This stress may trigger changes in animal foraging behaviour or affect their ability to fight infections. So, how do researchers document how an animal would behave if no one were watching? And more importantly, how do wildlife researchers make sure that they have minimal effects on the animals they study?

Mark Ditmer and his team from the University of Minnesota set out to quantify the stress response in animals exposed to drones. Specifically, the team wondered whether black bears could get used to the presence of drones over time and if that tolerance could remain, even if drones only passed by every few months. To do this, the team implanted heart rate loggers into five captive American black bears and monitored changes in heart rate as a proxy for stress. In the spring, they flew a drone over the bear enclosure five times per day and two times per week for 4 weeks. These same flights were then repeated in the fall to see if the bears exhibited the same stress response after not hearing a drone all summer.

As expected, on the very first drone flight, the bears’ heart rates increased by about 50 beats per minute. However, over time, this increase got smaller. By the third week of drone flights, the bears had become used to the drone noise. What's more, when the drone flights started back up again in the fall months, the bears did not exhibit the large increases in heart rate like they did in the spring! This means that the bears were accustomed to the drone noise: they no longer exhibited a strong stress response even though they had not heard a drone in 3 months!

Nowadays, bears and other wildlife are frequently exposed to drone noise, so it is reassuring to know that bears can habituate to the noise in just a few weeks. This finding can help researchers be confident that their observations are representative of true wild behaviours, at least in American black bears. However, this does not mean drones are completely harmless. For example, getting used to sounds of machines may make animals less wary of cars, increasing the risk posed by roads. Drones represent a great new technology that can help many different conservation efforts, but good intentions do not absolve humans from being conscientious about our impacts on wildlife.

**Cite as:** Ditmer MA, Werden LK, Tanner JC, Vincent JB, Callahan P, Iaizzo PA, Laske TG, Garshelis DL (2019) Bears habituate to the repeated exposure of a novel stimulus, unmanned aircraft systems. *Conservation Physiology.* 7(1):coy067.

Illustration by Erin Walsh; Email: ewalsh.sci@gmail.com